# Updates on Pathophysiology of Discogenic Back Pain

**DOI:** 10.3390/jcm12216907

**Published:** 2023-11-02

**Authors:** Rohan Jha, Joshua D. Bernstock, Joshua I. Chalif, Samantha E. Hoffman, Saksham Gupta, Hong Guo, Yi Lu

**Affiliations:** 1 Harvard Medical School, Boston, MA 02115, USA; 2 Department of Neurosurgery, Brigham and Women’s Hospital, Boston, MA 02115, USA

**Keywords:** discogenic back pain, low back pain, IVD degeneration, pathophysiology

## Abstract

Discogenic back pain, a subset of chronic back pain, is caused by intervertebral disc (IVD) degeneration, and imparts a notable socioeconomic health burden on the population. However, degeneration by itself does not necessarily imply discogenic pain. In this review, we highlight the existing literature on the pathophysiology of discogenic back pain, focusing on the biomechanical and biochemical steps that lead to pain in the setting of IVD degeneration. Though the pathophysiology is incompletely characterized, the current evidence favors a framework where degeneration leads to IVD inflammation, and subsequent immune milieu recruitment. Chronic inflammation serves as a basis of penetrating neovascularization and neoinnervation into the IVD. Hence, nociceptive sensitization emerges, which manifests as discogenic back pain. Recent studies also highlight the complimentary roles of low virulence infections and central nervous system (CNS) metabolic state alteration. Targeted therapies that seek to disrupt inflammation, angiogenesis, and neurogenic pathways are being investigated. Regenerative therapy in the form of gene therapy and cell-based therapy are also being explored.

## 1. Introduction

Chronic back pain is a common medical problem that imparts a notable socioeconomic health burden. The prevalence is around 30%, though up to 80% of individuals report having some form of back pain at least once in their life [1]. It is the most common cause of disability and years lived with disability worldwide [2], with the United States spending 100 billion dollars in lower back pain-associated annual healthcare expenditures [3]. The etiologies of lower back pain are multifactorial and include disc herniation; spinal canal stenosis; facet joint osteoarthritis; myofascial or spontaneous causes; and discogenic back pain [4]. Discogenic back pain, which specifically arises from intervertebral disc (IVD) degeneration, is the most common cause globally, comprising anywhere from 26–42% of cases in individuals with chronic lower back pain [5]. Large epidemiological studies have provided more robust evidence that IVD degeneration is significantly associated with low back pain [6,7,8]. If magnetic resonance imaging (MRI) findings of IVD exist, individuals are 2–3× more likely to experience back pain than those individuals without MRI evidence of IVD [9].

Discogenic back pain is pain specifically arising from IVD degeneration and excludes other etiologies of low back pain, including radicular pain from disc herniation, anatomical deformities, and other etiologies identified above. Moreover, since pain implies the presence of innervation, discogenic back pain can be more appropriately thought of as pain originating from damaged IVD due to the irritation of nerves that supply the damaged disc [10]. Though degeneration is associated with discogenic back pain and is thought to be an important contributing factor, degeneration by itself does not imply discogenic back pain; in fact, the absence of degeneration is more specific for the lack of discogenic pain [11]. However, if degeneration does exist, the MRI grading of disc degeneration using the Pfirrmann grade or Modic criteria does not correlate with severity or even the presence of discogenic back pain [5]. Rather, it is likely that persistent inflammation and the subsequent innervation drives symptomatic degenerated intervertebral discs [12].

Though the pathophysiology of discogenic back pain is incompletely understood, here, we aim to identify the biomechanical and biochemical steps that contribute to its development in the setting of degenerative disc disease.

## 2. IVD Anatomy

**Figure 1:**
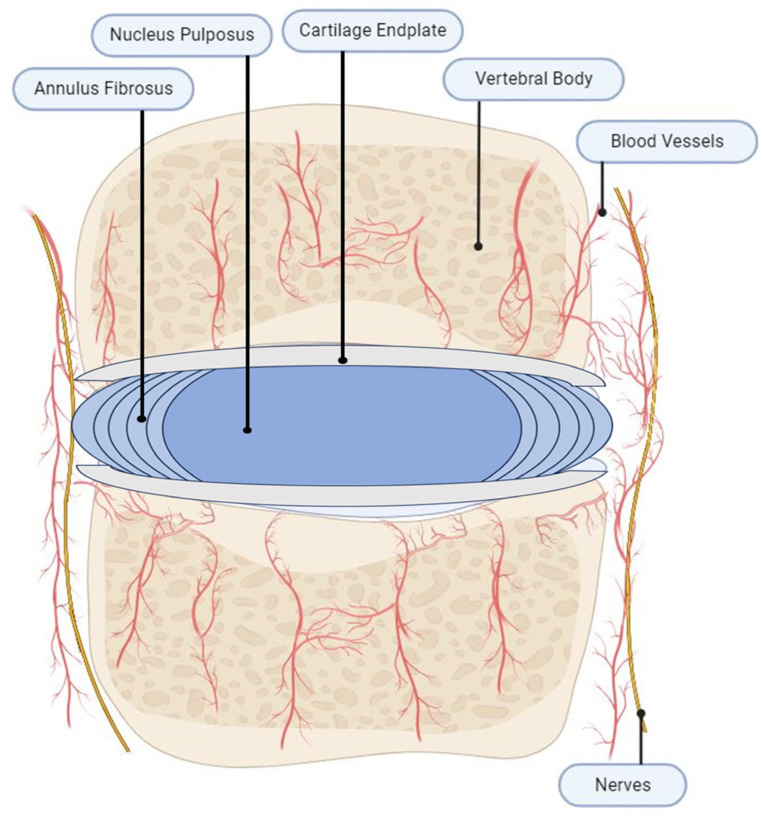
Healthy IVD. A healthy intervertebral disc (IVD) has three main components: the inner nucleus pulposus (NP), the outer annulus fibrosus (AF), and the cartilaginous endplates (CEPs). The CEP anchors the disc to the adjacent vertebrae. The healthy NP is a gelatinous and proteoglycan-rich tissue and enclosed by AF. AF is a highly organized fibrous structure composed of concentric lamellae of tilted collagen fibers with scattered proteoglycans. Arteries supply the outermost region of AF, while sinuvertebral nerves innervate the outer third of the AF. Relatively minimal neural innervation and vascularization exists in the healthy state. NP is avascular and aneural.

Intervertebral discs (IVDs) are cartilaginous joints located between each vertebra that are essential for the alignment, stability, and mobility of the spine [13,14]. The IVD is composed of the inner nucleus pulposus (NP), surrounded by an annulus fibrosus (AF), and is limited superiorly and inferiorly by the cartilaginous endplates (CEP) [13]. The NP is comprised primarily of water, but also includes proteoglycans, collagen fibrils, and elastin [14], which allows it to attract water to effectively distribute hydraulic pressure and maintain disc height. Large notochordal cells and mesenchymal cells are the main cellular components of the NP, which differentiate during aging and are progressively less capable of self-maintenance [15]. The AF is composed of not only organized concentric collagen fibers that surround the NP core but also water, proteoglycans, and elastin [14], which permits resistance to circumferential loading. The AF is subdivided into the inner AF, composed of type II collagen fibers and chondrocyte-like cells, and the outer AF, composed of type I collagen fibers and fibroblast-like cells in a series of concentric lamellae [16]. The CEP is located above and below the IVD, attaching the discs weakly to the vertebrae. It is composed of water, type II collagen, and proteoglycan, and functions to provide nutrient–waste exchange [17] (Figure 1).

In the normal, non-degenerated state, the production and degradation of the IVD extracellular matrix (ECM) are balanced. Relevant degradative enzymes and catabolic agents in IVD homeostasis include matrix metalloproteinases (MMPs), “a disintegrin and metalloproteinase” (ADAMS), Interleukin 1 (IL-1), and Tumor Necrosis Factor (TNF-α), and relevant growth factors include bone morphogenic protein-2 (BMP-2), BMP-7, Growth Differentiation Factor 5 (GDF-5), Transforming Growth Factor-b (TGF-β), Insulin-like Growth Factor-1 (IGF-1) [18,19]. Pathologic degeneration occurs due to a mismatch between these catabolic and anabolic processes, as well as the insufficiency of steady-state metabolism [5].

## 3. Intervertebral Disc Damage and Degeneration

### 3.1. Risk Factors for Degeneration

Degeneration is thought to be a natural consequence of aging, but environmental and genetic risk factors can accelerate this process [20]. Risk factors that have been identified for IVD degeneration include aging, mechanical loading, vibration exposure, inadequate nutrition, genetic predispositions, smoking status, trauma history, ineffective microorganism clearance, and metabolic syndrome [4,20,21]. Obesity has a positive correlation between early disc generation and discogenic pain severity. A possible hypothesis is the elevated serum levels of IL-6, C-Reactive Protein (CRP), TNF-α, and leptin in obesity may induce inflammatory response in the IVD [22]. Smoking has been found to both induce and worsen the severity of existing discogenic back pain. Possible mechanisms to explain this association include the reduction in blood flow to the IVD [23]. The genetic heritability of IVD degeneration is estimated to be between 34% and 61%, with mutations, polymorphisms, and complex inheritance mechanisms accounting for this variability [24]. In addition, epigenetic contributions have also been identified in playing an essential role in the progression of human disc degeneration. A recent study carried out a genome-wide DNA methylation profile of human degenerated NP tissues, and found significant clusters and difference in methylation profiles of the early and late stages of degeneration [25].

### 3.2. Process of IVD Degeneration

The pathophysiology of IVD degeneration involves a variety of biochemical and biomechanical processes including inflammation, cellular exhaustion and phenotype changes, and ECM degradation [26]. Degeneration likely begins with a reduction in NP notochordal cells with aging, which normally function in upregulating matrix synthesis pathways and downregulating degradative proteases. The loss of notochordal cells would therefore result in the global reduction in the anabolic pathway activity corresponding to a lower IVD regenerative capacity [26,27]. Furthermore, cellular exhaustion is associated with the loss of proteoglycans and glycosaminoglycans, which transition the NP from an elastic, hydrated state to a more fibrous one [28,29]. In the AF, the native cells are replaced with cartilage-like cells, and the concentric organized structure becomes fractured [30]. Finally, the loss of notochordal cells is also associated with increased inflammatory mediators such as IL-1β, IL-6, Intercellular Adhesion Molecule 1 (ICAM-1), Lymphocyte function-associated antigen 1 (LFA-1), Fibroblast Growth Factors (FGF) and nerve growth factor (NGF) [31,32].

Alongside cellular reduction, the IVD begins accumulating biomolecular damage, including free-radicals, advanced glycation end products, and epigenetic damage that results in oxidative stress [33]. Diabetic rat models of IVD have also shown increased advanced glycation end products, reduced IVD glycosaminoglycan content, increased CEP thickness and sclerosis, and decreased CEP porosity [34]. The accumulation of advanced glycation end-products induces ectopic calcification, CEP decellularization, and the production of pro-inflammatory cytokines from the NP [35]. Glycation end-products have also been associated with the increased expression of hypoxia-inducible genes and oxidative stress [34], which in turn can lead to subsequent NLR family pyrin domain containing 3 (NLRP3) inflammasome-mediated pyroptosis in NP cells [36].

As a result of these multifactorial initial insults, the disc responds with further cellular senescence, apoptosis, and dysregulated signaling. Significant increases in senescent and apoptotic cells have been seen in degenerating IVD. They begin secreting cytokines, chemokines, proteases, and growth factors which all contribute to the inflammatory milieu in the degenerating IVD [20]. Multiple apoptotic pathways have been identified that correlate to the severity of IVD degeneration. In the early stages, the endoplasmic reticular pathways are active. In the advanced and severe stages, the death receptor and mitochondrial pathways are active, respectively [37]. Apoptosis leads to a worsening inflammatory microenvironment as the debris is unable to be cleared efficiently in the avascular space.

**Figure 2:**
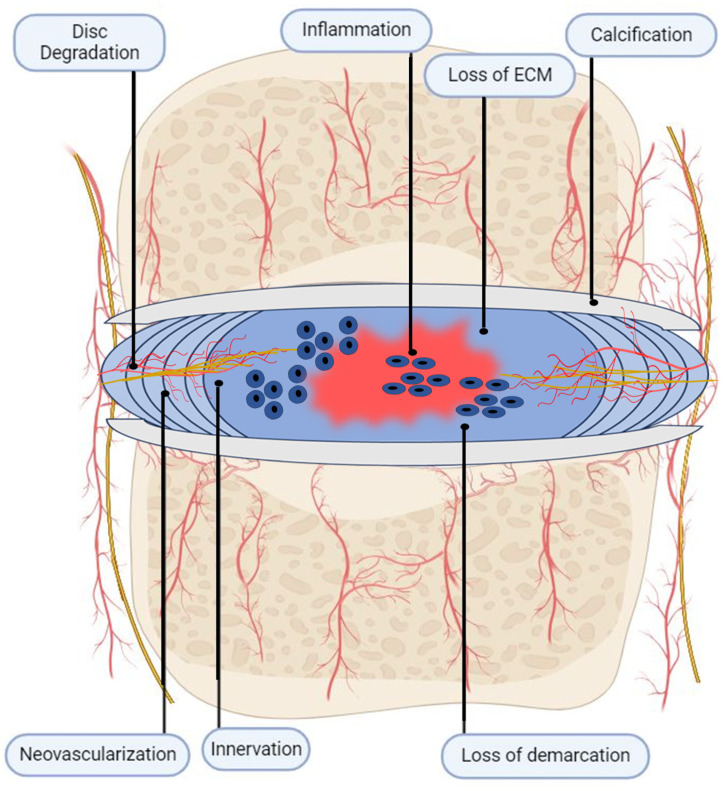
Degenerated IVD. Degenerative IVD is characterized by inflammatory milieu and loss of ECM architecture matrix. The structural integrity of the disc is lost, as fissures begin to occur, affecting tissue biomechanics. Blood vessels and nerve fibers are presented in the inner region of AF and NP as a result of aberrant healing and repair. Structural changes in the IVD, including annular bulging and osteophyte formation in the CEP, are usually present.

These changes produce a degenerated IVD characterized by reduced hydration, elasticity, disc height, mechanical integrity, and load-bearing capacity (Figure 2). Most importantly, microscopic structural damage such as tears and fissures begin to emerge [17,38,39]. As degeneration continues, osteophyte formation, reduced CEP permeability, and denser NP tissue develop, obstructing nutrient–waste exchange [40]. Tissue hypoxia and lactic acidosis results, generating an acidic microenvironment within IVDs that further compromises disc integrity [41] and promotes peripheral nociceptive sensitization [42], as discussed in the sections below. Through these mechanisms, the degenerative cycle of the IVDs continues to perpetuate itself as the disc continues to lose its matrix integrity, cell populations, and native biomechanics. However, until neovascularization and pathological innervation occur in response to degeneration, discogenic back pain does not occur.

## 4. Inflammatory Activation in Degenerated IVDs

### 4.1. Initial Inflammatory Activation

With IVD degeneration, the concentrations of inflammatory mediators increase in the IVD and peri-discal space due to the simultaneous upregulation of the inflammatory response within the IVD as well as increased recruitment from circulating cells [20]. In the disc itself, endogenous crystal and ECM breakdown product deposition may initiate the inflammatory response [43]. The phagocytosis of these deposits, such as calcium pyrophosphate, magnesium whitlockite, and hydroxyapatite, can trigger the NOD-like receptor family pyrin domain containing 3 (NALP3) inflammasome, which triggers the maturation and release of IL-1β [44]. Additionally, the byproducts of ECM degradation may trigger inflammation. Fibronectin end-products have been shown to promote monocyte infiltration [45]. Elastin, laminins, collagen, thrombospondin, and hyaluronan fragments have all also been associated with cytokine production and inflammatory activation [46]. The fragments of hyaluronic acid can activate IL-1β, IL-6, IL-8, Cyclooxygenase-2 (COX-2), and metalloproteases in human IVD cells [47].

In response to the increase in inflammatory mediators, the cells within the NP and AF begin producing higher levels of pro-inflammatory cytokines. IL-1β and TNF-α levels are known to increase with symptomatic disc degeneration and have been the most studied mediators in both mice models and clinical studies [44]. In addition, IL-6, IL-17, and Interferon gamma (IFN-γ) are all inflammatory mediators that are overexpressed in degenerated discs [20]. Other possible pro-inflammatory molecules such as nitric oxide, leukotrienes, prostaglandins, and lactic acid have all been observed in increased levels in the models of IVD degeneration [10]. NGF, which mainly functions in neoinnervation, can activate the Nuclear factor kappa-light-chain-enhancer of activated B cells (NF-kB) pathway to induce the production of multiple pro-inflammatory cytokines in IVD degeneration [48,49]. COX-2 has also been linked to inflammatory pathways accompanying discogenic pain [50]. In individuals with obesity, inflammatory markers such as IL-6, CRP, and TNF-α have been associated with the progression of discogenic back pain [51]. The levels of substance P (SP), a neuropeptide, increase in symptomatic degenerated IVDs, which further upregulates downstream chemokines and TNF-α in the AF, and IL-1β, IL-6, and IL-8 throughout the IVD [44,52].

### 4.2. Immune Cell Recruitment

Immune cells such as macrophages, neutrophils, natural killer (NK) cells, and lymphocytes are recruited to the degenerated IVD through the expression of chemokines and are allowed to infiltrate in part due to the loss of structural integrity [53]. TNF-α and IL-1β production by NP cells are associated with Th17 cell and macrophage recruitment [54]. Increased IL-6 levels in the NP also seem chemotactic for inflammatory CD14+ macrophages, but the chemotactic role of growth factors (NGF, vascular endothelial growth factor (VEGF)) remains controversial [55,56]. Mast cell migration, which is upregulated in painful IVDs, can induce an inflammatory, catabolic, and angiogenic state in NP cells. Mast cell-specific tryptase can upregulate VEGF-α to promote angiogenesis and degrade proteoglycans to create a catabolic microenvironment in the IVD [57]. Furthermore, they can augment additional inflammatory recruitment to the IVD via CCL2/MCP-1 chemoattracts. Of note, healthy IVDs have been shown to inhibit the activation of these mast cells [58].

A positive feedback cycle begins to establish itself, where the production of cytokines and chemokines further recruits immune cells [53]. In a rabbit lumbar model of disc degeneration, there were multiple peaks in inflammation markers, suggesting a long-term pro-inflammatory state [59]. Non-degenerated discs normally respond with the reduction in inflammatory gene expression if an inflammatory milieu is introduced, whereas degenerated discs enter a positive feedback cycle, suggesting that the homeostatic response is lost [60]. The inflammatory response likely continues to persist as the previously immune-privileged disc becomes a source of “non-self” molecules, thereby recruiting more immune cells [44].

### 4.3. Inflammation Stimulates Further IVD Degradation

The increased levels of inflammatory cytokines and chemokines have been known to stimulate ECM degradation enzymes while reducing the expression of ECM anabolic mediators in IVD degeneration [61,62,63]. Cytokines such as TNF-α, IL-1, and IL-6 have been shown to activate proteolytic enzymes including MMPs and a disintegrin and metalloproteinase with thrombospondin motifs (ADAMTS) [53,62], which play a major role in ECM degradation. MMP1, MMP2, MMP3, MMP9, MMP10, MMP13, MMP14, ADAMTS4, ADAMTS5, and ADAMTS7 expression is elevated in symptomatic degenerated IVDs in mice models, which is theorized to contribute to both matrix degradation and nociception induction [20,64]. These proteolytic enzymes likely modulate syndecan-4 (SDC4), and prolyl hydroxylases-3 (PHD-3), which act to promote the NF-κB pathway and ERK pathway, which further drive IVD degradation [20]. In addition, the overexpression of Osteopontin (OPN) and CD44 may also have a role in exacerbating ECM degradation [65]. Growth Differentiation Factor 5 (GDF5) has also been shown to promote ECM catabolism [66].

**Figure 3:**
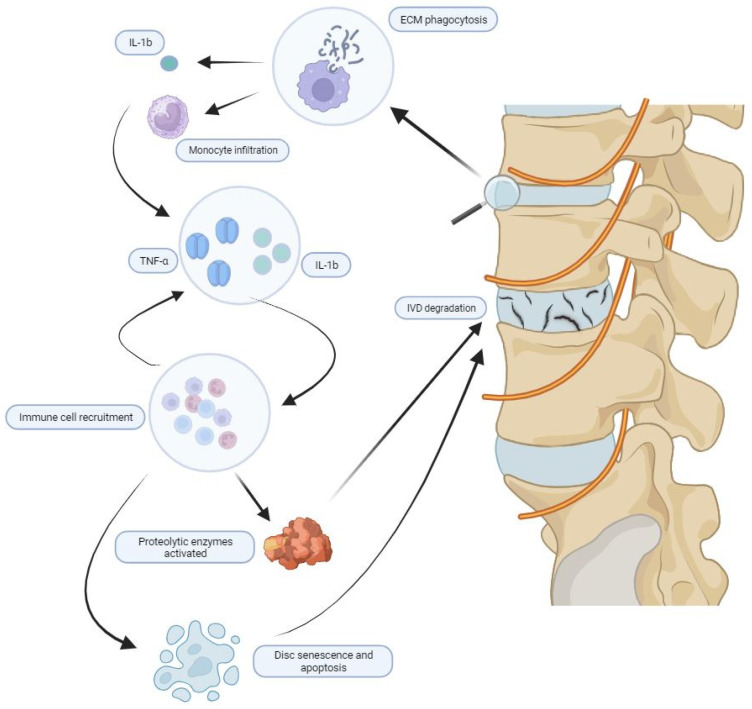
Overview of molecular pathways of degradation. Overview of molecular pathways of IVD degeneration. ECM fragment and crystals serve as initial insults. Phagocytosis of these materials leads to an upregulation of IL-1b and monocyte infiltration, which serve to upregulate inflammatory mediators, most notably, TNF-a and IL-1b. A positive feedback cycle emerges whereby these mediators lead to immune cell recruitment, which further upregulates these inflammatory mediators. Various proteolytic enzymes are activated, and the disc undergoes further senescence and apoptosis to facilitate IVD degradation.

Inflammatory mediators also contribute to deficiencies in native cell proliferation, disc senescence, and apoptosis. IL-1β and TNF-α promote the dysregulation of Notch signaling pathways, decreasing cellular proliferation and differentiation [67], and therefore contributing to the inability of the degenerated IVD to repopulate itself with cells [68]. Senescent cells in degenerating discs secrete TNF-α, IL-1β, IL-6, proteases, and chemokines that promote the further senescence of nearby cells [69]. The expression of Calcitonin gene-related peptide (CGRP) is increased in degenerated tissue, which, through the NF-kB and Mitogen-activated protein kinases (MAPK) pathways, has been known to inhibit the proliferation and induce the apoptosis of NP cells [70]. IL-1β and TNF-α have also been shown to promote apoptosis via miRNA in both AF and NP cells and may induce cellular autophagy in degenerating discs [71,72]. Through stimulating the production of other inflammatory mediators, recruiting additional inflammatory cells, and enhancing matrix degradation, cytokines and chemokines produced from IVD degeneration lead to the progression of degeneration (Figure 3).

## 5. Neovascularization and Aberrant Healing

Despite the inflammatory nature of the IVD degenerative cycle, discogenic back pain does not occur until the neovascularization and pathological innervation stages of degeneration. The IVD is mainly avascular and thus relies on the movement of oxygen, nutrients, and waste through the CEP and dissemination into the disc via diffusion [13,73]. During degeneration, inflammation and hypoxia destroy the ECM, and fissures begin emerging in the AF. The attempted repair of these fissures leads to vascularization and the formation of granulation tissue, which is hypothesized to initiate discogenic pain based on animal model data [5,74].

The aberrant healing and neovascularization in the NP are mediated by the mass expression of growth factors from the inflammatory exudate. VEGF, Basic Fibroblast Growth Factor (bFGF), TGF-β, Pleiotrophin, and Connective Tissue Growth Factor (CTGF) all facilitate endothelial cell migration to form new capillary networks in areas of active repair [30,75,76,77], bFGF likely has an important role in cell division and proliferation acceleration. TGF-β likely acts directly on fibroblasts and also functions in the formation of granulation tissue [78,79]. Both of these factors are strongly expressed in the NP and AF of painful degenerative discs [80]. The increased levels of IL-8, which is highly pro-angiogenic, also likely contributes to the neovascularization [81]. Notably, asymptomatic degenerative discs do not have such mass expression of growth factors, vascular granulation tissue formation, or innervation [30].

In addition to growth factor expression, additional components of the degenerated IVD facilitate angiogenesis. Healthy NP cells and intact aggrecans impede migration and induce the apoptosis of vascular endothelial cells [82]. Hence, the loss of proteoglycans during disc degeneration decreases the capacity of the AF to inhibit neovascularization [83]. Inflammation from disc degeneration leads to the downregulation of Tissue Inhibitor of metalloproteinase-3 (TIMP3) in NP cells, alleviating the suppression of angiogenesis that may therefore promote multiple pathways for the development of discogenic pain [84]. The expression of NGF in degenerated IVDs also serves as a chemotactic agent for endothelial cells [85].

**Figure 4:**
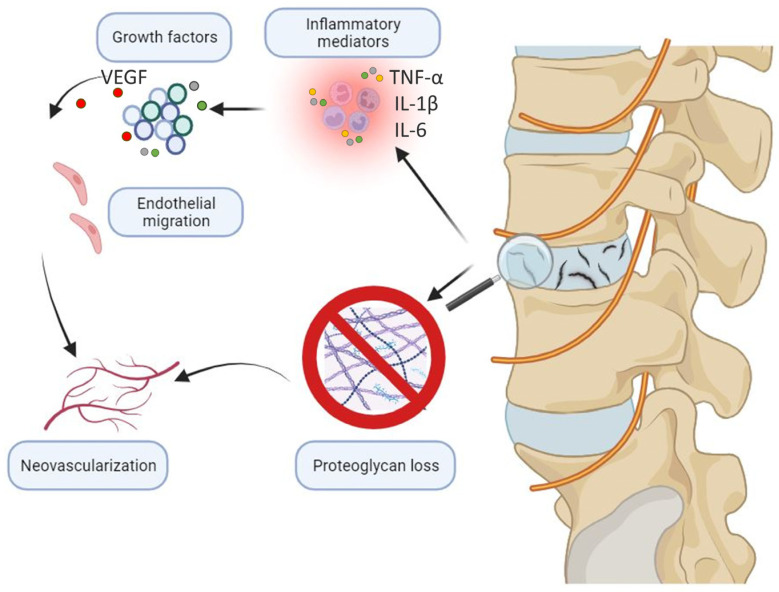
Overview of neovascularization in discogenic back pain. Overview of neovascularization in IVD degeneration. The hypoxic and inflammatory microenvironment of degenerated IVD enhances the ingrowth of blood vessels. Inflammatory microenvironments destroy ECM and mediate vascular endothelial cell migration. Inflammatory factors, such as tumor necrosis factor (TNF)-α, interleukin (IL)-1β, and IL-6 stimulate the expression of VEGF in IVD cells, thus accelerating the process of IVD neovascularization.

Though these factors otherwise contribute to growth, regeneration, and healing, they are likely inciting pathological changes in the disc in an attempt to repair it. Importantly, the emergence of new microvessels in degenerating IVDs is usually accompanied by neoinnervation, as they likely provide both nutrition and mechanical tracts for axon growth [86]. Capillaries have been known to grow with nerves through endplate injury/disruption [87]. This pathological innervation is likely the key mediator for the onset of discogenic back pain (Figure 4).

## 6. Pathological Innervation and Sensitization

### 6.1. Innervation of the Non-Degenerated IVD

In the non-degenerated state, the IVD has limited neural innervation, with the outer AF and endplate having minimal innervation and the NP normally lacking innervation completely [88]. Small diameter dorsal root ganglia (DRG) supply the sensory fibers that innervate the IVD [89]. The sensory fibers that do innervate the disc are mainly nociceptive, though there are components of proprioceptive fibers. In addition, sympathetic vasomotor afferents may contribute to the transmission of pain signals in the non-degenerated IVD. These nerves may be accompanied by blood vessels or may exist alone as free nerve endings [90].

### 6.2. Inflammation and Neovascularization Promotes New Nerve Growth

Degeneration and its inflammatory sequelae lead to nerve growth into the AF and NP, thereby expanding the distribution of nociceptive nerve endings [91]. The total number of nerve fibers in the disc increases, and there is an extension of sensory nerves and sympathetic afferents into normally aneural areas [92,93]. Notably, the profile of this pathological innervation is identical to that of a normal disc; while the type of innervation does not change, the quantity and scope of innervation changes in patients with discogenic pain [94]. New nerve growth was more commonly associated with fissures or damaged areas within painful IVD and mostly comprised of sinuvertebral nerve and basivertebral nerve fibers [88,95]. The expression of proteins associated with axon generation and nociceptive neurotransmitters initially suggested the presence of pathological innervation [92]. Further evidence for innervation is from the increased expression of Protein Gene Product 9.5 (PGP 9.5), a neuron marker [96], and Growth Associated Protein 43 (GAP43) protein in AF and NP tissue from individuals with discogenic back pain [4]. As highlighted below, this neoinnervation is likely the most pathological mechanism of discogenic back pain, and likely is the most significant differentiator between symptomatic and asymptomatic degenerative discs.

### 6.3. Neurotrophins (NTs) Induce New Nerve Growth

Neurotrophins (NTs) act to promote the survival, proliferation, chemotaxis, and differentiation of neurons [97]. Neurons innervating the IVD consistently express Tropomyosin receptor kinase A (TrkA), TrkB, and Ret, which are receptors for NTs [10]. In a non-degenerated IVD, NTs such as NGF and Brain-derived neurotrophic factor (BDNF) are expressed at variable levels, though in generally low or undetectable quantities [98,99]. However, following IVD degeneration and inflammation, NTs are massively upregulated. The increased levels of NTs and their receptors have been observed in IVDs with symptomatic degeneration, suggesting a correlation between NT expression and innervation density in IVD [96]. Inflammatory cytokine production from degenerating discs, most notably IL-1 and TNF-α, induce increased expression levels of NTs such as NGF, BDNF, NT-3, and NT-4/5 from the disc, which in turn recruit nerve fibers via the activation of the receptors Trk and p75NTR [77,96,97]. 

Beyond the degenerating IVD, inflammatory cells such as eosinophils, mast cells, lymphocytes, macrophages, and new microvessels are all additional sources of NTs [49,87,100]. Other stimuli for the increased production of NGF in degenerative discs include IL-1β, TNF-α, Platelet-derived growth factor (PDGF), IL-4, IL-8, and TGF-β [101]. The newly proliferated nerve themselves have been known to produce additional neuropeptides, such as substance P, that further react to NGF and perpetuate nerve growth ingrowth [87,96]. In total, IVD cells, neurons themselves, and inflammatory infiltrate can all serve as sources of NTs.

Additional mechanisms beyond NTs may also explain neoinnervation in degenerating discs. Netrin-1 has also been known to promote both axon growth and vascular endothelial cell migration, whereas its blockage reduced neoinnervation, angiogenesis, and spinal hypersensitivity [102]. Semaphorin 3a is natively expressed in high levels in healthy discs and likely functions in inhibiting infiltrative neural growth. Their levels decrease in degenerative discs and hence may have a contributory role in neural ingrowth [103]. In addition, the high levels of proteoglycans in the native disc maintain the absence of innervation. As the levels of proteoglycans, most notably aggrecans, decrease, there is a corresponding loss of negatively charged moieties, leading to an absence of innervation suppression [82].

### 6.4. Nociceptive Sensitization Occurs in IVDs

In addition to promoting the ingrowth of sensory nerves, NTs have a role in nociceptive sensitization, i.e., altering the sensory nerves to a more hypersensitive and inflammatory state [104]. Sensory neuropeptides and ion channels in neurons participate together in this process to create heightened sensory nociceptive transmission. NGF changes gene expression within DRG neurons to facilitate the expression of neuropeptides such as SP and CGRP. Furthermore, it facilitates the insertion of calcium, potassium, Transient receptor potential vanilloid 1 (TRPV1), P2X purinoceptor 3 (P2X3), and proton ion channels in the membranes of both terminal and DRG neurons [10,105,106]. Sodium channels such as NaV1.7 have also been known to be upregulated in DRG neurons innervating degenerated symptomatic discs. Antibody suppression of the sodium channels shows decreased levels of CGRP [107]. NGF also has a role in expressing acid-sensitive nociceptive ASIC receptors, where increased microenvironment acidity from lactic acid accumulation in the degenerated disc can increase nociceptive pain [108]. All of these channels are associated with ischemic and inflammatory pain and are consistent with nociceptive signaling. Glial Cell Line-derived Neurotrophic Factor (GDNF) has been shown to be constitutively expressed in human IVD cells, with enhanced expression in a pro-inflammatory and degenerative microenvironment [109]. With properties similar to NGF, the expression of GDNF also likely plays a role in hyperalgesia and neuronal sensitization.

In addition to upregulation, the functional alterations of these receptors may also mediate the nociceptive adaptive response [110]. Finally, NTs may also act directly on nociceptive fibers to trigger responses. NGF activates the p38 MAPK pathway to amplify and sensitize nerve action potentials [111]. BDNF has been shown to modulate signals in both the DRG and innervating fibers of the IVD [112].

Nociceptive sensitization in neurons may occur independently of NTs and can involve other molecular triggers. TRPV1 is functionally altered in DRG neurons of in vitro models of degenerated discs, independently of NGF levels [110]. Furthermore, VEGF may also have a direct role in pain transmission in IVD degeneration. VEGF receptor-deficient mice showed decreased discogenic pain in response to degenerative disc disease induction; these mice expressed lower levels of pain factors and were also associated with lower spinal glial activation [113]. Hypoxic and acidotic conditions are capable of causing spontaneous and bradykinin-stimulated sensitization of the dorsal root ganglion [114]. In addition, sympathetic nerves may contribute to peripheral sensitization as they increase their response to inflammatory, pressure, or ischemic changes [89]. Neurons are also sensitized to mechanical loading in the degenerated IVD; normally innocuous mechanical stimuli can generate amplified nociceptive responses, leading to peripheral sensitization. Mechanoreceptors in the outer AF and polymodal nociceptors respond to mechanical stimulus, cytokines, protons, and other inflammatory mediators [4], which has been shown to increase neuronal hypersensitivity in sensory afferents in mice models of disc degeneration [115]. The increased nociceptive sensitivity to physiologic mechanical stimuli is likely mediated by elevated IL-6 levels [116].

The inflammation of nerves with cytokines produced by IVD cells, immune infiltrates, and the surrounding milieu contributes to hypersensitization. These mediators act directly on nervous nociceptors, increasing the likelihood of action potential generation and therefore contributing to hyperalgesia [117,118,119]. For instance, inflammatory mediators such as TNF-α, IL-1β, IL-1, IL-6, IL-8, and the CCL/CXCL/CX3CL family increase the production of sensory neuropeptides and increase the activity of membrane ion channels on the surface of axons [104,120]. In addition, microglial cell and astrocyte activity are upregulated in intervertebral disc degeneration, which assists in a pro-neuroinflammatory state by inducing both cytokines and neurotrophic factors [104]. As a result, the hypersensitive state of afferent nerves is maintained, and there is a lower threshold to produce nociceptive nerve impulses.

Nociceptive sensitization occurs both at the level of the disc as well as the spinal cord to facilitate discogenic pain [4]. Afferent neurons project signals to the dorsal horn of the spinal cord to convey nociceptive information via different fiber types. NGF promotes the production and retrograde transport of nociceptive NTs and neuropeptides such as substance P, CGRP, and BDNF to the dorsal horn of the spinal cord [121,122]. Furthermore, with prolonged inflammation, afferent neurons in the dorsal horn increase the sensitivity to fibers from peripheral discogenic fibers [4]. The increased sensitivity is primarily due to input fiber phenotype switching, where innocuous stimuli peripherally lead to an exaggerated central response [123]. In addition, NGF-induced BDNF expression appears to modulate small-diameter sensory neurons at the level of the dorsal horn [112]. In summation, symptomatic IVDs are characterized by nociceptive sensitization at the neuron level due to the increased expression of NTs, inflammatory milieu, and microenvironment changes. In addition, multiple pathways exist to induce sensitization at the level of the spinal cord as well.

## 7. Emerging Pathophysiology

There is some evidence that disc infection with low virulence organisms may contribute to symptomatic disc degeneration and symptomatic low back pain, with an OR ~6 [124]. *Propionibacterium (Cutibacterium) acnes* was observed to be the most common contributor in a meta-analysis identifying low virulent organisms in human symptomatic IVDs [124]. However, another case–control trial showed no difference in the bacterial growth of disc samples from case and control groups of degenerated IVDs [125]. A meta-analysis of clinical studies of individuals treated with chronic courses of antibiotics demonstrated improved low back pain outcomes [126]. While the mechanism underlying infection-mediated symptomatic degeneration remains unknown, existing data implicates the release of proinflammatory cytokines and the production of exocellular virulence factors [124]. Recent evidence with rat models with *P. acnes* inoculation showed the overexpression of SP, CGRP, IL-1β, IL-6, and IL-8 via the TLR2/NF-kb p65 pathway from NP cells induced discogenic back pain [127,128]. *P. acnes* was also associated with the upregulation of NGF expression via the TLR2-NF-kB/JNK pathway, which may contribute to the discogenic pain observed in rat models [129].

Other emerging pathophysiology may contribute to the schema described herein so far. Discogenic pain may also lead to alterations in the metabolic state of the CNS. Chronic back pain leads to the accumulation and activation of microglia in the spinal cord and the brain [129]. Increased colony-stimulating factor signaling likely mediated the activation and accumulation of these microglia in symptomatic degenerated discs [130,131]. Hormonal interactions may also alter the inflammatory cascades involved in the pathogenesis of discogenic back pain. Mouse models with ovariectomies show an increased expression of inflammatory cytokines and neuropeptides such as TNF-α, IL-1β, IL-6, SP, and neurokinin 1 receptor (NK1R) in NP cells, whereas estrogen replacement therapy was able to reverse the upregulation. Estrogen was thus theorized to protect against disc degeneration via a reduction in pro-inflammatory cytokines as well as a reduction in SP [121]. Finally, abnormally expressed miRNAs have been found in degenerative intervertebral discs [132]. miRNAs may induce NP cell apoptosis as well as abnormal proliferation, AF degeneration, ECM degradation, and the induction of inflammatory factors to induce IDD [133].

There is newly emerging evidence that Colony Stimulating Factor 1 Receptor (CSF1R) signaling in microglia/macrophages plays a central role in the development of both IVD degeneration and discogenic back pain [134]. In my lab, we found that the deletion of CSF1R from microglia/macrophages or oral administration of CSF1R competitive inhibitor, GW2580, ameliorated proinflammatory cytokines release after intervertebral disc injury, which led to decreased IVD degeneration. Additionally, the deletion of CSF1R in microglia/macrophages prevented microglia activation within spinal cord and macrophage activation in DRG and blocked neuropathic pain generation and centralization. These modifiers in the IVD experimental model culminated with a reduction in IVD degeneration and neuropathic pain, and thus could hold future promise in the development of therapeutic modalities. A summary of the major mediators is identified in the table below (Table 1).

Though this review has primarily focused on the molecular mechanisms of discogenic back pain, the importance of psychopathological co-factors in the development of this pain pattern cannot be understated. The influence of psychosocial factors has been increasingly characterized in generalized low back pain, where these factors are known to influence both the manifestation, severity, and chronicity of low-back pain, while also impacting outcomes after treatment [21,135,136]. Though there are limited investigations specifically with respect to discogenic back pain, established findings in low back pain are likely highly applicable to discogenic back pain.

## 8. Therapeutic Approaches

Traditional approaches for discogenic back pain initially involve conservative management with analgesics or NSAIDS, followed by steroid injections and surgical fusion if refractory. However, as these approaches focus on treating the pain symptom or changing the biomechanical nature of the spine, rather than seeking to re-establish the native physiological and biomechanical disc properties [21], they are often are limited in their efficacy. Conservative treatments include physical therapy and exercise, but their effects are generally limited and effective only in mild cases, and the escalation of treatment is often required. Discogenic back pain that is refractive to conservative measures may then be treated with epidural anesthetic or steroid injections with moderate efficacy [137]. Though the mechanism underlying this effect is not fully characterized, these injections likely interrupt nociceptive sensory inputs and temporarily reduce the presence of inflammatory mediators [137,138]. Delivery of a local anesthetic to the L2 nerve root has also provided relief from discogenic back pain [139], as has radiofrequency ablation of basivertebral and sinuvertebral nerves, albeit in a small cohort [95]. DRG stimulation has also improved chronic discogenic back pain in a small cohort [140]. As an absolute ultima ratio, surgical disc removal and fusion can be pursued, especially in cases of CEP-based discogenic back pain [141]. Ultimately, surgical fusion cannot restore either the structure or function of affected IVDs [142].

### 8.1. Emerging Targeted Therapies

Targeted therapies that disrupt the pro-inflammatory, angiogenic, and neurogenic pathways or that support tissue regeneration may lead to the successful resolution of discogenic back pain [44]. Biologic treatments that have been explored include gene therapy, stem cell therapies, and bioengineered tissues; however, they have been mostly limited to the preclinical stages [38].

Approaches targeting specific inflammatory mediators and new vessel/nerve growth are emerging as promising agents for discogenic pain, with development ranging from in vitro experiments to clinical trials. Injections of TNF-α blockers and IL-1 inhibitors in close proximity to the IVD have shown some success in reducing back pain [44,119,143]. Notably, TNF-α blockade did not reverse matrix-degrading activity, suggesting that its role is likely more localized to nerve root irritation and discogenic pain production [44]. IL-1Ra delivered via microspheres have shown the sustained attenuation of IL-1β and its downstream cytokine mediators in vivo [144], but their effects of directly decreasing discogenic back pain in humans have yet to be characterized. IκB Kinase-β may simultaneously suppress TNF-α, IL-1β, and IL-6 in the disc, and neuropeptides in DRG neurons, to reduce discogenic back pain [145]. NF-kB antagonists in rabbit degenerated IVDs show decreased IL-1 and TNF-α expression, along with a reduction in the pain response [146]. Intradiscal injections of small molecule antagonists of CCR1 and CCR2 were effective in blocking THP-1, a human monocytic cell line, migration and splenocyte migration, respectively, in vitro; however, their effects in reducing inflammatory marker expression were limited to a couple of weeks [147]. In addition, as mentioned above, the CSF-1R antagonist holds great promise in reducing IVD degeneration and discogenic back pain [134].

In addition to inflammatory antagonists, therapies targeting new blood vessel or nerve growth may also prove efficacious in reducing discogenic pain. For example, VEGF inhibitors have been shown to be effective in reducing inflammatory cytokines in rat models [148]. Ion channel inhibitors, such as the ASIC3 inhibitor APETx2, CBS inhibitor AOAA, and anti-NaV1.7 improved pain hypersensitivity behavior in rat models [104]. Anti-NGF antibodies have also been evaluated in randomized control trials (RCTs), which showed analgesic efficacy but have limitations with tolerability [149,150,151]. RCTs in humans showed intradiscal methylene blue injections are effective in alleviating discogenic back pain, as they destroyed nociceptors and alleviated the inflammatory response [152,153].

The limitations of intradiscal injections modalities include the short half-life of acting molecules, the limited effects from targeting limited pathways in an otherwise complicated degenerative/nociceptive system, and the potential for the paroxysmal worsening of IVD degeneration by puncturing the IVD [20]. Conversely, systemic delivery is unlikely to reach therapeutic doses in IVD due to the limited vascular supply and diffusion limitations of the degenerated disc [44].

### 8.2. Emerging Regenerative Therapies

Gene therapy and cell-based therapies may allow for prolonged analgesic effects and regenerative strategies. Methods that have been explored include gene transfer via viral vectors, gene silencing via RNAi, and gene editing via CRISPR/CAS-9 [154]. The transduction of genes for TGF-b3, CTGF, and TIMP metallopeptidase inhibitor 1 (TIMP1) in a rabbit model resulted in increased aggrecan and collagen in degenerated discs [155]. The limitations of gene therapy include high dose exposure, misplaced injections, and the possibility of oncogenesis. Increased reliability in vector constructs and transgene expression will be required [156].

Current cell-based clinical strategies to treat discogenic pain include mesenchymal stem cell (MSC)/bone marrow aspirate therapies, platelet-rich plasma (PRP), and chondrocytes. MSC therapy is likely the best candidate as this allows for autologous transplantation and multiple cell-type differentiation. The intradiscal injection of MSC shows an improvement in discogenic pain and functionality via stimulating ECM regeneration, downregulating pro-inflammatory cytokine production, and providing analgesic effects, but its therapeutic effects may be limited [20,157,158]. Autologous bone marrow aspirate concentrates injected intradiscally produced improvement in chronic discogenic low back pain for at least one year [159]. One case report demonstrated that umbilical cord-derived MSC injection into the bloodstream, lumbar epidural facet joints, and epidural space generated acute improvement in back pain [160]. Co-administration with ortho-vanillin may enhance this therapeutic effects through increased proteoglycan synthesis, decreased IL-6 and IL-8 release, and a decreased number of senescent cells [161]. The large-scale safety and efficacy of MSC injection has yet to be demonstrated [162], and its exact immunomodulatory mechanism of action remains unclear; future study is required to determine the role of cell-based therapy in patients with discogenic back pain [20].

Platelet-rich plasma (PRP) has also been shown to reverse chondrocyte degeneration in intervertebral discs [163]. A recent clinical trial in sixteen patients with discogenic back pain showed improved disability scores following PRP administration, but similar pain and quality of life levels when compared to corticosteroid injections [164]. Similarly, chondrocyte transplantation studies show initial efficacy in reducing degeneration in adult patients, but larger-scale studies are required to determine efficacy [165].

Finally, NP and AF repair or replacement using synthetic biomaterials remain areas of active research. Tissue engineering strategies to regenerate entire discs are also being developed [166]. Given the avascular nature of the native IVD, and worsening cellularity with degeneration, regenerative strategies would likely be most effective earlier in the course of degeneration [167]. Hyaluronic acid hydrogel implantation in mice models was associated with the downregulation of nociceptive markers, decreased hyperinnervation, and decreased inflammatory activation to reduce nociceptive behavior [168]. A summary of the therapeutic approaches to discogenic back pain is identified in the table below (Table 2).

## 9. Conclusions

Discogenic back pain results from the degeneration of the IVD, but IVD degeneration is not sufficient to induce pain. Though the pathophysiology of discogenic back pain remains incompletely understood, a likely framework suggests that degeneration leads to inflammatory activation and immune cell recruitment. As a result of chronic inflammation, neovascularization, neoinnervation, and nociceptive sensitization follows, which ultimately manifests as discogenic pain. Identifying the nuances of these biochemical pathways will be paramount in developing targeted therapeutic approaches that seek to ameliorate discogenic back pain.

## Figures and Tables

**Table 1:** Summary of major mediators involved in pathogenesis of discogenic back pain.

Initial IVD Degeneration	Cellular Loss
	Oxidative stress products: free radicals, advanced glycation end products
	Apoptotic pathways
Initial Inflammatory Activation	ECM breakdown phagocytosis → NALP3 → IL-1β
	Fibronectin end products, hyaluronic acid fragments
	IL-1β, TNF-α
	IL-6, IL-7, IFN-γ
	NGF → NF-kB
	SP
Immune Cell Recruitment	IL-1β, TNF-α
	IL-6
	Mast Cells
Persistent Inflammatory Degradation	MMPs
	ADAMTS
	SDC4, PHD-3 → NF-kB, ERK
	OPN, GDF5
	IL-1β, TNF-α → positive feedback senescence
	CGRP → NF-kB, MAPK
Neovascularization	VEGF
	bFGF, TGF-β, CTGF, IL-8, Plieotrophin
	Loss of proteoglycans, ↓ TIMP3
Pathological Innervation	NTs (NGF, BDNF, NT-3, NT-4/5)
	Netrin-1, Semaphorin 3a
Nociceptive Sensitization	NGF → SP, CGRP, Ion Channels, ASIC receptors
	GDNF, BDNF
	VEGF
	Mechanoreceptors
	Inflammatory cytokines
Emerging Pathophysiology	Low virulence infections
	Microglia metabolic activation
	miRNAs
	CSFR1

**Table 2:** Therapeutic approaches to discogenic back pain.

Current Therapies	Conservative Management	Analgesics
		Physical Therapy and Exercise
	Invasive Measures	Epidural Anesthetics
		Steroid Injections
		Dorsal Root Ganglion Stimulation
		Surgical Discectomy and Fusion
Emerging Therapies	Targeted Therapies	TNF-α blockers
		IL-1 inhibitors
		NF-kB antagonists
		VEGF inhibitors
		Anti-NGF antibodies
	Regenerative Therapies	Gene Therapy
		Mesenchymal Stem Cell Therapy
		Platelet-Rich Plasma Therapy
		Tissue Engineering Strategies

## Data Availability

Not applicable.
